# Investigation of a nonsense mutation located in the complex KIV-2 copy number variation region of apolipoprotein(a) in 10,910 individuals

**DOI:** 10.1186/s13073-020-00771-0

**Published:** 2020-08-21

**Authors:** Silvia Di Maio, Rebecca Grüneis, Gertraud Streiter, Claudia Lamina, Manuel Maglione, Sebastian Schoenherr, Dietmar Öfner, Barbara Thorand, Annette Peters, Kai-Uwe Eckardt, Anna Köttgen, Florian Kronenberg, Stefan Coassin

**Affiliations:** 1 Institute of Genetic Epidemiology, Department of Genetics and Pharmacology, Medical University of Innsbruck, Schöpfstrasse 41, A-6020 Innsbruck, Austria; 2 Department of Visceral, Transplant and Thoracic Surgery, Medical University of Innsbruck, Innsbruck, Austria; 3 Institute of Epidemiology, Helmholtz Zentrum München - German Research Center for Environmental Health (GmbH), Neuherberg, Germany; 4 German Centre for Cardiovascular Research (DZHK), Partner Site Munich Heart Alliance, Munich, Germany; 5 Department of Nephrology and Hypertension, Friedrich-Alexander Universität Erlangen-Nürnberg, Erlangen, Germany; 6 Department of Nephrology and Medical Intensive Care, Charité—Universitätsmedizin Berlin, Berlin, Germany; 7 Institute of Genetic Epidemiology, Faculty of Medicine and Medical Center, University of Freiburg, Freiburg, Germany

## Abstract

**Background:**

The concentrations of the highly atherogenic lipoprotein(a) [Lp(a)] are mainly genetically determined by the *LPA* gene locus. However, up to 70% of the coding sequence is located in the complex so-called kringle IV type 2 (KIV-2) copy number variation, a region hardly accessible by common genotyping and sequencing technologies. Despite its size, little is known about genetic variants in this complex region. The R21X variant is a functional variant located in this region, but it has never been analyzed in large cohorts.

**Methods:**

We typed R21X in 10,910 individuals from three European populations using a newly developed high-throughput allele-specific qPCR assay. R21X allelic location was determined by separating the *LPA* alleles using pulsed-field gel electrophoresis (PFGE) and typing them separately. Using GWAS data, we identified a proxy SNP located outside of the KIV-2. Linkage disequilibrium was determined both statistically and by long-range haplotyping using PFGE. Worldwide frequencies were determined by reanalyzing the sequencing data of the 1000 Genomes Project with a dedicated pipeline.

**Results:**

R21X carriers (frequency 0.016–0.021) showed significantly lower mean Lp(a) concentrations (− 11.7 mg/dL [− 15.5; − 7.82], *p* = 3.39e−32). The variant is located mostly on medium-sized *LPA* alleles. In the 1000 Genome data, R21X mostly occurs in Europeans and South Asians, is absent in Africans, and shows varying frequencies in South American populations (0 to 0.022). Of note, the best proxy SNP was another *LPA* null mutation (rs41272114, *D*′ = 0.958, *R*^2^ = 0.281). *D*′ was very high in all 1000G populations (0.986–0.996), although rs41272114 frequency varies considerably (0–0.182). Co-localization of both null mutations on the same allele was confirmed by PFGE-based long-range haplotyping.

**Conclusions:**

We performed the largest epidemiological study on an *LPA* KIV-2 variant so far, showing that it is possible to assess *LPA* KIV-2 mutations on a large scale. Surprisingly, in all analyzed populations, R21X was located on the same haplotype as the splice mutation rs41272114, creating “double-null” *LPA* alleles. Despite being a nonsense variant, the R21X status does not provide additional information beyond the rs41272114 genotype. This has important implications for studies using *LPA* loss-of-function mutations as genetic instruments and emphasizes the complexity of *LPA* genetics.

## Background

High lipoprotein(a) [Lp(a)] plasma concentrations are a major risk factor for cardiovascular diseases (CVD) in the general population [1–9]. Fifteen to 25% of the population present Lp(a) concentrations above 30–50 mg/dL that put them at increased cardiovascular risk [2, 10]. Unlike other lipoproteins, more than 90% of Lp(a) variance is controlled by a single gene locus [11] named *LPA*, which encodes the distinctive structural protein of the Lp(a) particle, the apolipoprotein(a) protein [apo(a)].

This highly repetitive protein contains several so-called kringle-IV domains (KIV-1 to KIV-10), where the KIV-2 domain is encoded in a coding copy number variation (CNV) that creates > 30 different apo(a) alleles (and thus isoforms) in the population [1]. The isoform size is inversely correlated with Lp(a) concentrations [1], with low molecular weight (LMW) apo(a) isoforms (≤ 22 KIV domains) being associated with 5–10-fold higher median Lp(a) concentrations than high molecular weight (HMW) isoforms (> 22 KIV domains) [1]. Interestingly, up to 40% of all individuals express only one isoform in the plasma despite being heterozygous on the DNA level. Some of these “null alleles” are due to nonsense mutations [12, 13] and/or inefficient secretion of overly large alleles [1], but additional genetic variants and mechanisms may play a role [14]. Finally, the genetics of Lp(a) is additionally complicated by the fact that the minor allele frequencies (MAF) of many single nucleotide polymorphisms (SNPs) in *LPA* show pronounced differences between ethnicities [15–17]. This is resembled also by the Lp(a) concentrations, which show a pronounced inter-ethnic variance, with individuals of African descent showing about five times higher median Lp(a) than Caucasians and a large variability within Asia [18]. Even within Europe, Lp(a) concentrations vary by at least twofold [19, 20]. On the individual level, Lp(a) concentrations can vary by about 100-fold even within the same ethnicity and still by 200-fold between individuals presenting indeed even the same isoform combination [21]. The reasons for this variance are largely unknown but have been shown to be mostly genetically determined [11, 21, 22].

Genotyping and sequencing in *LPA* are complex. Up to 70% of the coding sequence [23] is located in the KIV-2 repeat, which is not accessible by common sequencing and genotyping technologies. It is thus unknown how many functional variants are hidden in the KIV-2 region and what their contribution in determining the variance in Lp(a) levels is. Accordingly, it is also unknown whether KIV-2 variants are captured by available genome-wide association studies (GWAS) on Lp(a) [24–27] at least indirectly via linkage disequilibrium (LD) or not.

Recently, an ultra-deep next-generation sequencing (NGS) approach with a customized bioinformatic analysis pipeline allowed us to catalog the variation within the KIV-2 region [17]. Among several hundred variants [17], this revealed also a splice site variant (G4925A [23]) that is found in 20% of the population, is associated with an Lp(a) reduction of up to ≈ 30 mg/dL, and explains a considerable fraction of the individuals carrying an LMW isoform but presenting low Lp(a) concentrations (a hitherto puzzling aspect of the relationship between Lp(a) concentrations and apo(a) isoform size). We showed that G4925A is likely the causal variant underlying the GWAS hit rs75692336 [24].

A second likely causal SNP in the KIV-2 region is the nonsense mutation KIV-2 R21X (named g. 61 C>T in [13], 640 C>T in [17]), which leads to a truncated protein that is rapidly degraded [13]. Parson et al. [13] identified it by a laborious cloning approach [13], but it has never been explored further in large epidemiological studies, and its contribution to the Lp(a) levels in the population is unknown. This makes it an attractive candidate to explain some of the peculiarities of Lp(a), and it might have been simply hidden in plain sight until now due to its location in the KIV-2.

We developed an allele-specific TaqMan PCR assay (ast-PCR) targeting the R21X variant, as well as the previously described KIV-2 variant G4925A [23], and assessed the effect of R21X on Lp(a) concentrations in nearly 11,000 individuals. To link R21X to available GWAS datasets, we then used genome-wide SNP data from the German Chronic Kidney Disease (GCKD) study to assess the LD of R21X with SNPs outside the KIV-2 region. Given that in heterozygous individuals the effect size of a functional *LPA* SNP depends from the size (and thus the expression level) of the allele on which the SNP is located (high-expressing LMW allele or low-expressing HMW allele), we determined the allelic location of R21X by pulsed-field gel electrophoresis (PFGE). Finally, to put our findings in a broader perspective, we assessed the frequency of R21X in the 1000 Genomes (1000G) Project phase 3 sequencing data and determined the LD of R21X with its best tagging SNP from GCKD in all twenty-six 1000G populations.

## Methods

### Populations

Our study involved 10,910 individuals from three populations, namely GCKD [28] (German Chronic Kidney Disease), KORA F3 [29], and KORA F4 [29]. Informed consent was obtained from each participant, and the studies were approved by the respective Institutional Review Boards. Details on the studies are given in Table 1 and in Additional file 1: Supplementary Methods. In brief, KORA F3 and KORA F4 are two independent studies initiated both by the KORA (Cooperative Health Research in the Augsburg region) Initiative. They represent two non-overlapping samples drawn from the general population living in the region of Augsburg, Southern Germany. KORA F3 has been conducted in 2004/2005 and evolved from the WHO MONICA (Monitoring of Trends and Determinants of Cardiovascular Disease) study. The KORA F4 survey is a non-overlapping study sample drawn in the years 2006/2008. GCKD is an ongoing prospective observational study of 5217 Caucasian patients with moderately severe chronic kidney disease at enrollment that were recruited at nine institutions in Germany.

**Table 1:** Descriptive statistics

	GCKD	KORA F3	KORA F4
*n*	4771	3099	3040
Sex (F)	1896 (39.7%)	1602 (51.7%)	1576 (51.8%)
Age (years)	53, 63, 70	46, 57, 67	44, 56, 67
Age (F) (years)	51, 63, 69	47, 57, 67	44, 56, 67
Age (M) (years)	55, 64, 70	46, 58, 68	45, 57, 68
Lp(a) (mg/dL)	5.0, 11.6, 33.7	5.0, 11.2, 29.2	5.2, 11.7, 30.2
Lp(a) in LMW carriers (mg/dL)	25.7, 53.2, 77.9	18.3, 49.4, 72.6	23.7, 46.8, 66.5
Lp(a) in HMW carriers (mg/dL)	3.8, 8.6, 17.8	4.0, 8.8, 16.7	4.1, 9.0, 17.3
eGFR (mL/min/1.73 m^2^)	37.0, 46.0, 57.0	76.1, 89.1, 99.2	77.4, 89.3, 100.1
Total cholesterol (mg/dL)	176.5, 207.1, 239.1	191.0, 216.0, 242.0	188.0, 214.0, 240.0
HDL-C (mg/dL)	39.2, 48.2, 61.1	46.0, 56.0, 69.0	45.0, 54.0, 65.0
LDL-C (mg/dL)	89.2, 113.7, 142.7	105.0, 126.0, 148.0	112.0, 134.0, 158.0
Triglycerides (mg/dL)	117.6, 168.0, 238.7	88.0, 135.0, 200.0	71.5, 104.0, 150.0
Type 2 diabetes mellitus	1713 (35.9%)	248 (8.1%)	241 (7.9%)
rs41272114 allele freq.	0.03	0.02	0.02

Continuous variables normally distributed are provided as mean ± standard deviation. Continuous variables non-normally distributed are provided as 25%, 50%, 75% percentiles. eGFR was estimated using the CKD-Epi equation [30]

*LMW* low molecular weight apo(a) isoforms, *HMW* high molecular weight apo(a) isoforms, *F* females, *M* males, *eGFR* estimated glomerular filtration rate, *Lp(a)* lipoprotein(a), *HDLC* high-density lipoprotein cholesterol, *LDLC* low-density lipoprotein cholesterol, *n* number

PFGE genotyping needs large amounts of buffy coat to prepare the required megabase-sized agarose plug DNA. Since these are not commonly available for population studies, samples from three other sample sets of the same ethnicity were used for the PFGE experiments. These were from the CAVASIC (Cardiovascular Disease in Intermittent Claudication) study [31, 32] (*n* = 9 R21X positive samples), from an ongoing collection of liver tissue specimens for Lp(a) research (IRB Medical University of Innsbruck, AN2015-0056) (*n* = 5; two thereof R21X positive) and from anonymous blood samples obtained from the blood bank of the University Hospital of Innsbruck, Austria (*n* = 2; 1 thereof R21X positive).

### ast-PCR for R21X typing

We designed an allele-specific triplex TaqMan PCR assay (ast-PCR) that selectively amplifies the mutant bases of R21X [13] and G4925A [23], as well as an amplification control amplicon located in *PNPLA3* (design illustrated in Additional file 1: Fig. S1). For the assay development, the R21X and the G4925A variant bases were introduced into pSPL3 plasmids containing one KIV-2 repeat [23] using the QuikChange II Site-Directed Mutagenesis Kit (Agilent Technologies, Santa Clara, CA, USA) with minor modifications (Additional file 1: Supplementary Methods). To provide an additional thermodynamic disadvantage to unspecific pairings [33], various base mismatches were introduced in the allele-specific primers on positions − 2 or − 3 (from the 3′ end), and the performance of different primer designs was tested on plasmid mixes mimicking mutation levels from 100 to 0% mutant fraction (Additional file 1: Fig. S2). The most specific primers were taken forward (Additional file 1: Fig. S2). Fluorescently labeled, locus-specific TaqMan probes were added to allow amplification detection in a high-throughput setting. An amplicon in *PNPLA3* served as positive amplification control to detect false-negative reactions due to PCR failure. Technical details are provided in Additional file 1: Supplementary Methods, Additional file 1: Table S1, and Additional file 1: Table S2. The assay was run on a 384-well Thermo Fisher (Waltham, MA, USA) QuantStudio 6 qPCR system. The R21X assay was validated both against ultra-deep NGS data from Coassin and Schönherr et al. [17] and a commercial castPCR assay (Thermo Fisher; as used in [23]) with a sensitivity of 0.2% mutant fraction (determined according to the manufacturer’s instructions on a NGS-validated sample). For validation, our assay was run on 376 samples from KORA F4, identifying 14 R21X carriers, which were all confirmed also by the commercial castPCR assay. The reproducibility was tested on 477 samples run in duplicates. Additionally, each 384-well qPCR plate (*n* = 34) contained the same positive control sample. A slightly modified ast-PCR protocol was used to genotype the gene alleles separated by PFGE (Additional file 1: Supplementary Methods).

### ast-PCR data analysis

**Fig. 1:**
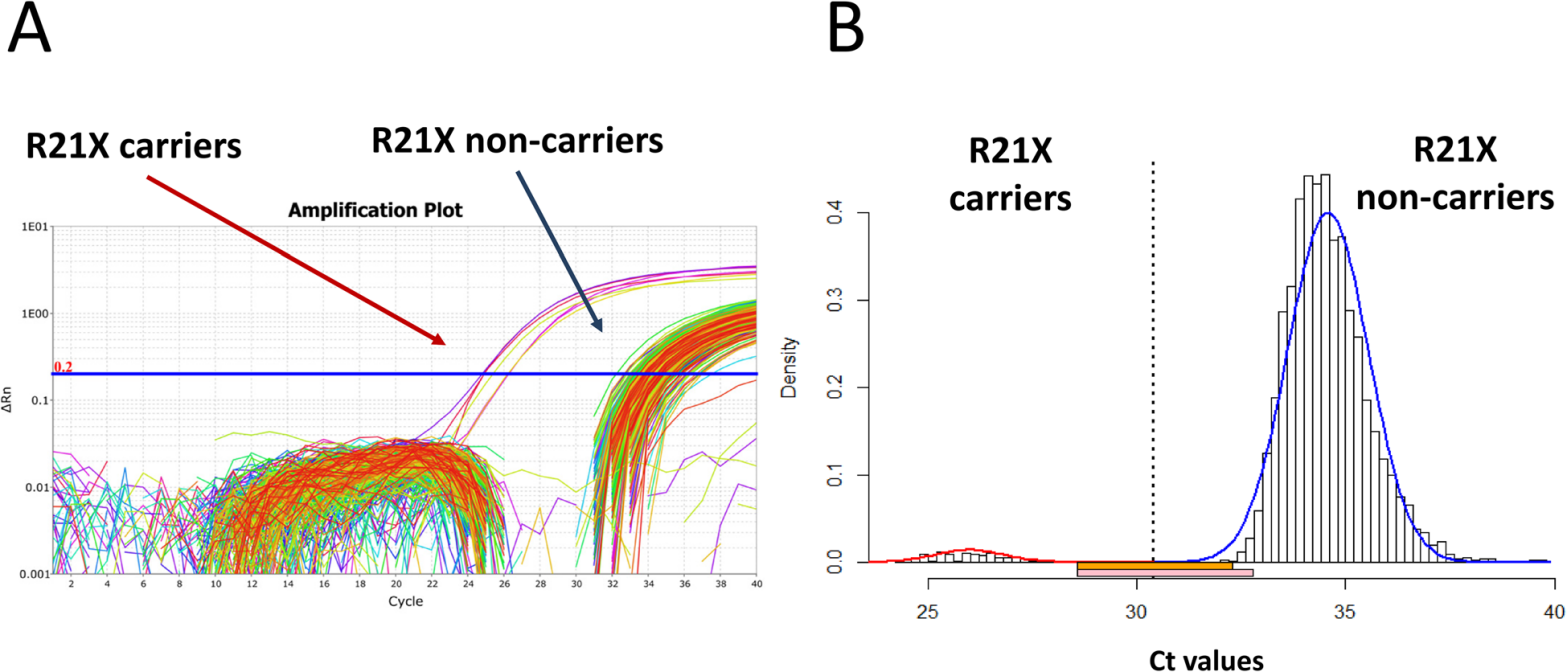
Example of the distributions of the Ct values in R21X carriers and non-carriers. **a** Exemplary ast-PCR amplification plot. **b** Discrimination of the two Ct distributions using a statistical clustering approach. The orange and pink horizontal bars below the *x*-axis identify the samples that cannot be uniquely assigned to one of the two distributions and are therefore excluded from the analysis. Orange bar: upper 1% of the carrier distribution and lower 1% of the non-carrier distribution. Pink bar: upper 2.5% of the carrier distribution and lower 2.5% of the non-carrier distribution (more conservative; used in this analysis). Plot generated using the provided R script

Figure 1 exemplifies the assay data analysis rationale. An unspecific amplification signal from a wild-type (WT) sample will occur later in the qPCR amplification than the true specific amplification of a mutant variant allele [Ct_(carriers)_ < Ct_(non-carriers)_]. This creates two Ct distributions whose widths are defined by stochastic fluctuation in the amplification of the target (e.g., due to the slightly varying input amount) and, for the mutant, the fraction of KIV-2 repeats affected (Fig. 1a). DNA input was 20 ng in all samples. Previous data [17, 23] indicates that the R21X mutant base is located on no more than one to three repeats, which translates to a maximum of ≈ 1.6 cycles difference due to the mutation level. To avoid human bias and have a systematic approach for sample assignment beyond pure visual clustering of the amplification curves, the optimal discrimination threshold between the Ct distributions of carriers and non-carriers was estimated using a bagged clustering algorithm [34] implemented in the R function *classIntervals* (package *classInt*), and two normal distributions were fitted to the two Ct distributions using the R package *VGAM* [35] (Fig. 1b). Details are provided in Additional file 1: Supplementary Methods. Samples that could not be assigned unambiguously to one of the Ct distributions (i.e., which could not be unambiguously identified as carriers or non-carriers) were excluded. The exclusion rate was 0.7% in GCKD (35/4974), 1.6% in KORA F3 (52/3157), and 0.5% in KORA F4 (15/3063). The R function used for analysis is available in our GitHub repository [36].

### Lp(a) phenotyping

All Lp(a) quantifications and apo(a) isoform determinations in all studies were performed in the same laboratory at the Institute of Genetic Epidemiology, Medical University of Innsbruck, Austria, using liquid handling robotics (TECAN, Männedorf, CH) with the same assay and evaluated by the same experienced researcher.

The Lp(a) concentrations and apo(a) isoforms were determined by ELISA and Western blotting as described previously [19, 37]. We used a polyclonal affinity-purified rabbit anti-human apo(a) antibody for coating and the horseradish peroxidase-conjugated monoclonal anti-apo(a) antibody 1A2 [38] for detection. Each sample was measured in a 1:150 and 1:1500 dilution, and the measurement which was in the optimal linear range of the OD reading of the 7-point standard curve was used for data analysis. In few samples with very high Lp(a) concentrations, we diluted the samples more than 1:1500. The lower detection limit of the assay is 0.1 mg/dL.

For Western blotting, 150 ng Lp(a) of each sample was loaded on a 1.46% agarose gel with 0.08% SDS (18 h, 0.04 A constant current). A size standard containing five plasma samples with only one apo(a) isoform of 13, 19, 23, 27, and 35 KIV repeats was applied in every seventh well of the gel. The gel was blotted semi-dry to a PVDF membrane and blocked with 1% BSA, 85 mM NaCl, 10 mM TRIS, 0.2% Triton X-100 (30 min, 37 °C). After incubation with horseradish peroxidase-conjugated 1A2 antibody, the membrane was washed, ECL substrate (WesternBright Chemilumineszenz Spray, Biozym, Vienna, AT) was added, and signals were recorded (Amersham Hyperfilm™ ECL™, GE Healthcare, Chicago, IL, USA).

### Identification of proxy SNPs

To allow linking R21X to existing GWAS results, we searched for a proxy SNP for R21X in the genome-wide SNP data of GCKD. Following the rationale that a SNP in LD with R21X will present a similar effect on Lp(a) and that this effect size should have been easily detected by our recent GWAS on Lp(a) (*n* = 13,781) [24], we created a contingency table of each of the 66 top hits of the isoform-adjusted model of our recent GWAS meta-analysis on the Lp(a) concentrations with the R21X [24] and analyzed it using Fisher’s exact test. We selected the two SNPs with the most significant *p* values (rs2489940, rs41272114) and calculated the LD in GCKD using the R package *genetics* [39]. The LD with rs41272114 was followed up further in KORA F3, KORA F4, and the 1000 Genomes data (see below). GCKD and KORA F3 and F4 were all imputed using the haplotype reference consortium (HRC) panel [40]. Imputation quality for rs41272114 was 0.948, 0.998 and 0.996, respectively.

### Pulsed-field gel electrophoresis

Based on the availability of suitable material, 12 samples positive for R21X were selected from the sample collections mentioned above for PFGE genotyping of *LPA* and were genotyped for rs41272114 using Sanger sequencing. Additionally, 4 non-carriers were added to be used as a negative control in the ast-PCR typing of the alleles isolated by KpnI PFGE (one being heterozygous for rs41272114).

PFGE genotyping [41, 42] was performed using two different enzymes. KpnI excises a region from KIV-1 to KIV-5 [41–43] and allows precise determination of the *LPA* allele size (estimated accuracy ± 1 KIV-2 repeat). The KpnI protocol was used to size the alleles and assess whether R21X is located on the large or the small gene allele (*n* = 12 carriers plus 4 non-carriers). Kpn2I digestion excises a much larger fragment that spans from *MAP3K4* to downstream of *LPA* [15] (> 600 kb in hg19; Additional file 1: Fig. S3). The Kpn2I protocol was used to assess also experimentally whether rs41272114 and R21X are located on the same haplotype (*n* = 12 carriers). Ten samples showed sufficient separation of the gene alleles to be used for haplotyping.

Technically, both PFGE protocols, Southern blotting, allele excision, and allele genotyping were done as described before [15, 23, 43]. In brief, agarose plug DNA has been prepared as described previously [15] and digested for 4 h at 37 °C (KpnI) or 55 °C (Kpn2I). Half plug for each sample was applied on the agarose gel twice and separated on a Bio-Rad (Hercules, CA, USA) CHEF Mapper system (Additional file 1: Table S3). One half of the gel was nicked by ultraviolet radiation and prepared for Southern blotting while the other half was kept native at 4 °C for allele excision and genotyping. Southern blot signal was detected using a DIG-labeled probe against KIV-2 hg19 chr6:161,054,945–161,056,154. The locations of the hybridization signals of the alleles were transferred to the other half of the gel, and the regions containing the alleles were excised from the gel [23, 43]. DNA was extracted from these gel slices using the peqGOLD Gel Extraction Kit (VWR, Radnor, PA, US). Genotyping was done using a modified ast-PCR protocol for R21X (see Additional file 1: Supplementary methods) and Sanger sequencing for rs41272114 (Additional file 1: Table S4).

### Frequency in 1000 Genomes data

We retrieved the frequency of R21X in 26 populations of the 1000 Genome (1000G) Project phase 3 from our recent catalog of genetic variation in the KIV-2 repeat [17] (named R20X there due to technical reasons [17]). In brief, all reads in the 1000G phase 3 [44, 45] high-coverage exome data that mapped to the *LPA* KIV-2 region (GRCh37; chr6:161,033,785–161,066,618) were downloaded as BAM file using *SAMtools* [46] and converted to FASTQ using *BEDtools2* [47]. These reads were then submitted to our *LPA Server* Pipeline [17, 23] (available in GitHub [48]). In this pipeline, all reads are aligned to one single repeat. Therefore, any mutation present in one or a few KIV-2 repeats is detected as low-level mutations present only in a fraction of reads [17, 23]. This resembles the calling of somatic mutation, but the *LPA* Server pipeline has been additionally adapted to cope with some peculiarities of the *LPA* KIV-2 sequence (described in [17]). A coverage of 340× and 780× at the position of R21X was required for high-confidence calls (i.e., the coverage limit for high confidence calling at ≥ 1% mutation level with the 95% confidence interval of a binomial distribution not crossing zero) in single and paired-end sequencing data, respectively [17].

The individual genotypes for rs41272114 in 1000 Genomes phase 3 were downloaded from Ensembl release 99. LD calculations were done using the R package *genetics* [39].

### Statistical methods

Differences in medians were assessed by the Wilcoxon test, and normality of continuous variables was tested with the Shapiro-Wilk test. The association between the *LPA* KIV-2 variant R21X and the Lp(a) levels was assessed by linear regression analysis in each population, adjusted for age and sex. GCKD analysis was also repeated with additional adjustment for estimated glomerular filtration rate (eGFR; estimated according to the CKD-EPI equation [30]) and urine albumin-to-creatinine ratio. R21X is a null *LPA* allele [13] and thus completely abolishes the respective isoform in the plasma. Since all remaining Lp(a) is thus produced by the non-mutant allele, the regression analysis was not adjusted for isoform, because this would imply to adjust for a major part of the Lp(a) concentration itself. *β* estimates were obtained on the original scale of Lp(a), while *p* value and coefficients of determination were derived after the inverse-normal transformation of the Lp(a) concentrations due to the skewed distribution. All analyses were done in the R software version 3.5.0 [49]. R package *metafor* [50] was used for fixed-effect meta-analysis.

## Results

### Assay performance

We established a cost-effective, high-throughput-capable ast-PCR for the simultaneous detection of carriers of the R21X variant [13] and G4925A [23] in large epidemiological sample collections. In this manuscript, we report the results for the R21X variant. The results of G4925A have already been reported earlier using a slightly different assay approach [23].

Our assay showed excellent sensitivity down to 0.5% mutant fraction and no amplification at 0% (Additional file 1: Fig. S2). The R21X assay also correctly classified six samples from our recent publication [17], where the R21X status had been previously determined by ultra-deep NGS (3 positive samples with mutation level 2.4–5.1% and 3 negative samples; all measured in triplicates; the number of positive validation samples was limited by the low carrier frequency). The Ct values ranged from 30.3 to 31.7 for the positive samples and from 37.3 to 39.7 for the negative samples, providing a clear separation between positive and negative samples. The validation of the assay against commercial castPCR (Thermo Fisher Scientific) showed no discordances (Additional file 1: Supplementary Methods). Reproducibility was tested by typing 477 GCKD samples twice during assay establishment and by typing 5–10% of the samples of each study twice (in total *n*_QC_samples_ = 879; for details, see Additional file 1: Supplementary Methods). No discordances were observed (Additional file 1: Table S5). The positive control sample gave the same result on each assay plate (*n* = 34). Sample call rates of the single studies ranged from 97.8 to 99.0% (Additional file 1: Table S5).

### R21X is associated with reduced lipoprotein(a) concentrations

**Fig. 2:**
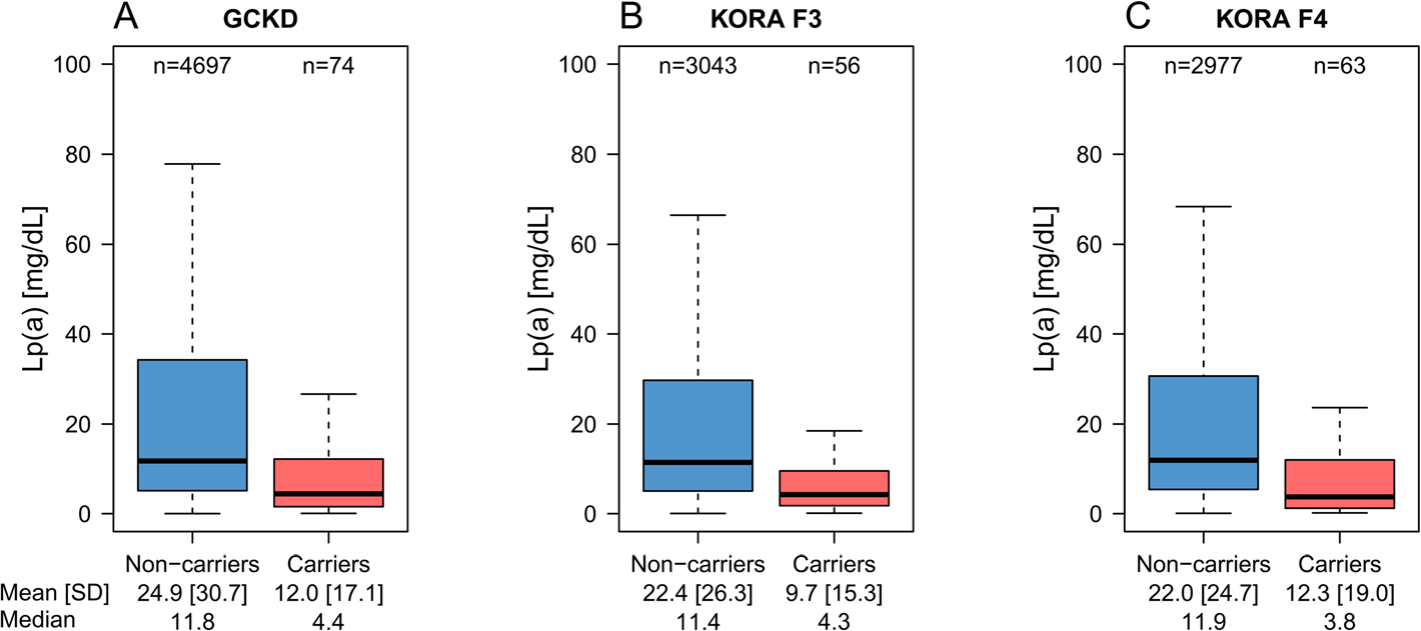
Association of the R21X variant with reduced Lp(a) levels. Lp(a) is lower in R21X variant carriers (i.e., at least one KIV-2 repeat carrying the R21X variant) in each population. Outliers are not shown to avoid an overly extended range of the scale due to the highly skewed distribution of Lp(a). The boxplots including the outliers are provided in Additional file 1: Fig. S5

We determined the R21X carrier status in 10,910 samples from three populations, two of them being population-based. The carrier frequency was 1.6% in GCKD, 1.8% in KORA F3, and 2.1% in KORA F4. Under the assumption of Hardy-Weinberg equilibrium, this translates to a minor allele frequency (MAF) of 0.78%, 0.91%, and 1.0%, respectively. The combined dataset contained 193 carriers. The R21X variant was associated with reduced Lp(a) levels in all three populations (Fig. 2, Table 2) with consistent effect estimates (Table 2). A fixed-effect meta-analysis resulted in an overall effect estimate of − 11.7 mg/dL (95% confidence interval (CI) − 15.5 to − 7.8; *p* = 3.39e−32). No heterogeneity was observed (Table 2). Adjustment for eGFR and urine albumin-to-creatinine ratio in GCKD altered the estimates only marginally (Table 2, footnote). Despite a MAF ≤ 1%, positive R21X mutation carrier status explained 1.1 to 1.5% of the variance of inverse-normal transformed Lp(a) (Table 2).

**Table 2:** Linear regression analysis between R21X carrier status and Lp(a) levels

Population	Carriers [***n***]	***n***	β*	95% CI lower bound*	95% CI upper bound*	SE*	***p***^**†**^	***R***^**2†**^	***I***^**2||**^
GCKD	74	4771	− 13.0^‡§^	− 20.0	− 6.0	3.6	4.34e−12	0.011	–
KORA F3	56	3099	− 12.6	− 19.5	− 5.7	3.5	6.50e−11	0.013	–
KORA F4	63	3040	− 9.9	− 16.0	− 3.8	3.1	3.95e−12	0.015	–
**Meta-analysis**	**193**	**10,910**	**− 11.7**	**− 15.5**	**− 7.8**	**1.9**	**3.39e−32**	**–**	**0.0**

Lp(a) concentrations are given as mg/dL. The *β* estimate is given on the original scale (mg/dL). Age- and sex-adjusted model

*n* number

*On the original scale

^†^On the inverse normal transformed scale

^‡^GCKD additionally adjusted for eGFR: beta = − 12.4 [− 19.58; − 5.25], SE = 3.7, *p* = 3.16e−10

^§^GCKD additionally adjusted for eGFR and urine albumin-to-creatinine ratio: beta = − 13.12 [− 20.22; − 6.02], SE = 3.6, *p* = 1.08e−10

^||^Heterogeneity was 0.0 on the original and on the inverse normal transformed scales

### R21X is located on moderately large alleles

We determined the identity and exact size of the *LPA* allele carrying the R21X mutation by KpnI-based PFGE (*n* = 12). The *LPA* alleles were separated, isolated from the gel, and genotyped separately for R21X carrier status. In all analyzed individuals, the R21X variant was located on HMW alleles in the range of 27–32 KIV. Under the null hypothesis that the SNP is equally distributed over all allele sizes and given the isoform size distribution observed in GCKD (27.5% of all 7328 alleles observed are located in this size range), the probability to observe such a distribution just by randomly sampling 12 individuals can be approximated as 0.275^12^ = 1.87 × 10^−7^. PFGE genotypes and the gene allele carrying the variant are reported in Additional file 1: Table S6.

### R21X is in linkage disequilibrium with the splice site variant rs41272114

We searched among the 66 top hits of our recent GWAS on Lp(a) [24] for a proxy SNP for R21X that would allow linking it to available GWAS data and thus determine whether any of the recently reported GWAS hits [24] picks up the signal of R21X. This identified two possible proxy SNPs: rs2489940 in *PLG* (MAF = 0.5%, *D*′ = 0.74, *R*^2^ = 0.38) and rs41272114 in *LPA* KIV-8 [12] (MAF = 2.6%, *D*′ = 0.958, *R*^2^ = 0.281). Both showed low *R*^2^ but high *D*′ values. The LD to rs41272114 was particularly noteworthy because rs41272114 itself is a widely studied splice donor mutation variant that causes *LPA* null alleles [12, 51, 52]. In KORA F3 and F4, *D*′/*R*^2^ were 0.96/0.286 and 0.932/0.31, respectively.

**Fig. 3:**
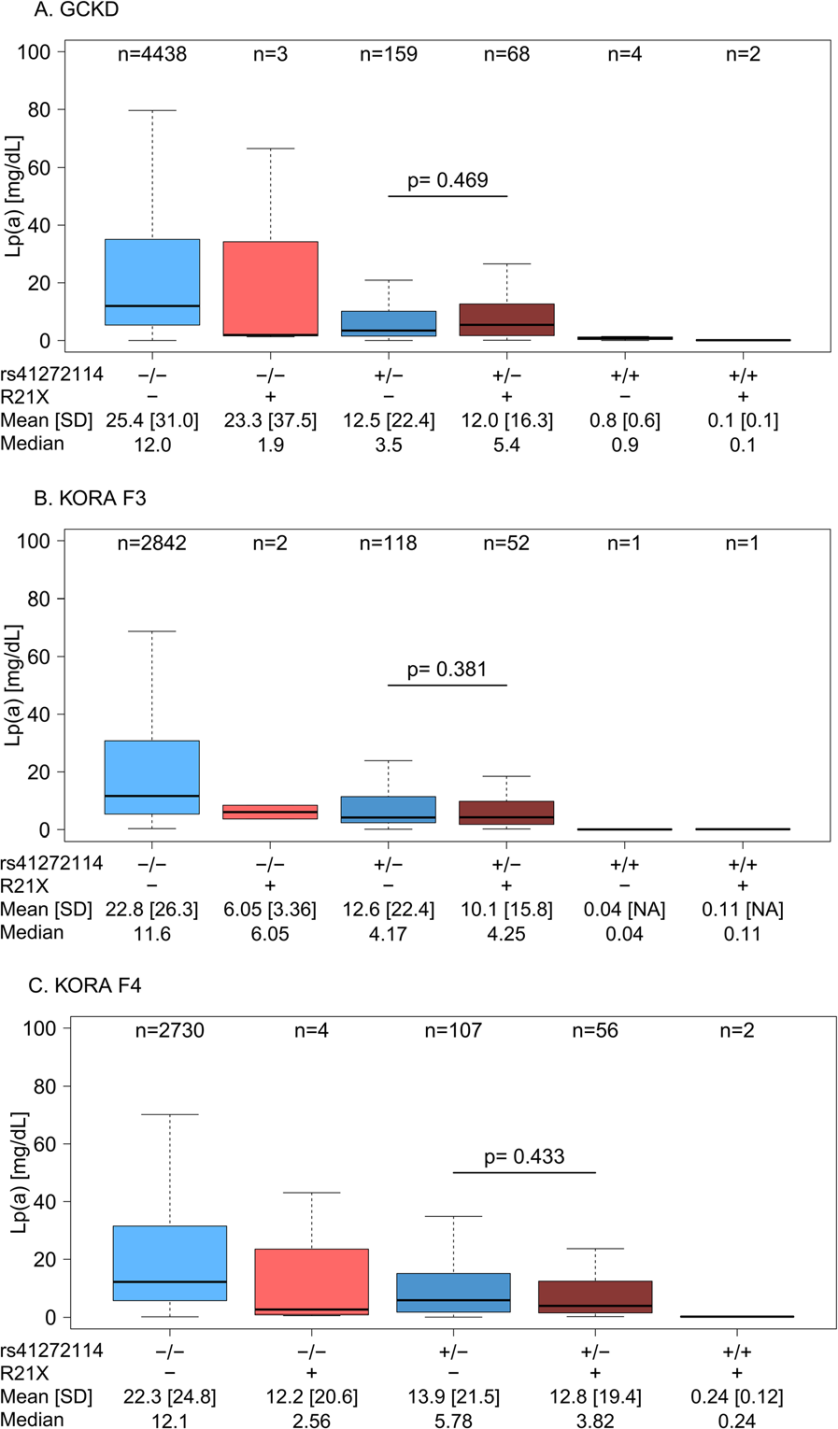
Lp(a) values in the carriers of the various combinations of R21X and rs41272114. No significant difference is observed between heterozygous individuals carrying only rs41272114 and those carrying both R21X and rs41272114. “+” indicates a minor allele. Rs41272114 is reported as genotypes, while for R21X, only carrier status (+ or −) is reported because most KIV-2 repeats still carry the wild-type base at any time, and thus, no narrow-sense genotype can be defined. No individuals homozygous for rs41272114 but wild type for R21X were observed in KORA F4. Note that the R21X-only group contains very few individuals in each population. *p* values assessed by the Wilcoxon test

The high *D*′ coefficient indicates that nearly all R21X carriers also carry rs41272114. To substantiate this experimentally, we re-run the PFGE protocol described above using Kpn2I instead of KpnI (*n* = 10 carriers). Kpn2I excises a genomic region that encompassed the complete genomic region from downstream of *LPA* to *MAP3K4* [15], which is located 500 kb upstream of *LPA* (Additional file 1: Table S4). Performing SNP genotyping on the separated alleles allows direct long-range haplotyping of any SNP within this region [15]. Indeed, in all samples tested, the R21X variant was located on the same gene allele as the rs41272114 variant. Accordingly, the association between R21X and Lp(a) vanished, if the linear regression model for R21X on Lp(a) in GCKD was adjusted for rs41272114 (*β* = − 0.67 (95% CI − 9.14; 7.81), *p* = 0.504, additionally adjusted for age, sex, and eGFR). Vice versa, rs41272114 was still associated with Lp(a) when the linear regression was adjusted for R21X (*β* = − 12.26 (95% CI − 17.00; − 7.55), *p* = 3.18e−29), respectively when linear regression was performed only in R21X-negative samples (*β* = − 12.26 (95% CI − 17.06; − 7.46), *p* = 3.90e−28). No significant difference in Lp(a) concentrations was found between heterozygous carriers of rs41272114 with and without R21X (Fig. 3a–c). Conversely, in samples that carried only R21X, the median Lp(a) concentrations were comparable to those of individuals carrying r41272114 alone (Fig. 3b, c).

### R21X is found mainly in European and South Asian populations

To extend our observations from Central Europeans also to other ethnicities, we retrieved the R21X genotypes from our recent catalog of KIV-2 variants in the 1000G Project [17] (Additional file 2). R21X was present mainly in Europeans (EUR, carrier frequency ≈ 0.024) and South East Asians (SAS, carrier frequency ≈ 0.019) (Table 3, Additional file 1: Table S7, Additional file 1: Table S8). It was not found in 1000G individuals of African ancestry despite this is the largest continental group (1000G super population) in the 1000G dataset. Only one carrier was observed in the East Asians (EAS) but did not fulfill the coverage requirements for the high confidence dataset (coverage > 780×). The carrier frequencies were remarkably robust to adaptions in coverage settings (Table 3, Additional file 1: Table S8), and the mutation levels (i.e., the fraction of KIV-2 carrying the mutation) were similar in all groups (Additional file 1: Fig. S4). While the allele frequencies of rs41272114 and R21X in EUR and SAS resembled the observations in our own dataset, a pronounced heterogeneity was observed in the group Admixed Americans from Middle and South America (AMR). Colombians (CLM) and Puerto Ricans (PEL) showed a similar R21X frequency as Europeans (1–2%), while R21X was not observed at all in Mexicans and Peruvians. Conversely, the MAF of rs41272114 varied very strongly and ranged from 3% in Colombians and Puerto Ricans to even 18% in Peruvians from Lima (PEL) (Additional file 1: Table S8). *D*′ was high in all super populations, while *R*^2^ varied widely (Table 3).

**Table 3:** Frequency of R21X and LD with rs41272114 in the 1000Gph3v5 super populations

	Super population				
	AFR	AMR	EAS	EUR	SAS
**All**					
Individuals [*n*]	661	347	504	503	489
Carriers [*n*]	0	3	1	12	9
Carrier frequency	0	0.009	0.002	0.024	0.019
LD rs41272114-R21X [*D*′/*R*^2^]	NA/NA	0.986/0.063	NA/NA	0.995/0.453	0.992/0.324
**Coverage > 340×**					
Individuals [*n*]	561	300	413	360	468
Carriers [*n*]	0	3	0	10	9
Carrier frequency	0	0.010	0	0.028	0.019
LD rs41272114-R21X [*D*′/*R*^2^]	NA/NA	0.986/0.068	NA/NA	0.995/0.387	0.992/0.347
**Coverage > 780×**					
Individuals [*n*]	548	279	332	272	468
Carriers [*n*]	0	3	0	7	9
Carrier frequency	0	0.011	0	0.026	0.019
LD rs41272114-R21X [*D*′/*R*^2^]	NA/NA	0.986/0.072	NA/NA	0.995/0.400	0.992/0.348

A coverage > 380× corresponds to a high accuracy variant call with single direction sequencing (single-end sequencing). A coverage > 780× corresponds to a high accuracy variant call using bidirectional confirmation (paired-end sequencing) and provides the most accurate variant calls. The rationale for these cutoffs is explained in the “Methods” section of this manuscript

Both the unfiltered data and the R21X carriers filtered for 340- and 780-fold coverage are shown

*AFR* Africans, *AMR* Admixed Americans, *EAS* East Asians, *EUR* Europeans, *SAS* South Asians, *LD* linkage disequilibrium, *n* number

## Discussion

The advent of potent Lp(a)-lowering agents [53] has renewed the interest in genetic variation in *LPA* [14, 52, 54–57]*.* Genetic variants in *LPA* have recently been used as a tool to estimate the amount of Lp(a) lowering that is likely required to produce a clinically meaningful CVD reduction [54, 55, 58]. Conversely, loss-of-function mutations in *LPA* have also been used to assess the safety of pharmacological Lp(a) reduction [53, 59] by using the effects of life-time low Lp(a) as a proxy for the effects of pharmacological Lp(a) reduction [52] (a concept known as genetic target validation [60]).

However, the genetic regulation of Lp(a) is intricate and consists of a complex interplay of the apo(a) isoforms and SNPs [23, 61]. Moreover, LD structures in *LPA* are partially restricted only to certain ethnicities [15, 62], and the isoform-Lp(a) relationship, as well as the median Lp(a) concentrations linked with a given isoform, vary between ethnicities [18] and even within the same ethnicity [18–20]. These factors require careful evaluation of *LPA* SNPs used as genetic instruments.

Despite the KIV-2 region contains a large part of the coding sequence of *LPA*, variants in the KIV-2 region have been neglected in *LPA* genetics for a long time, because they were not accessible. The introduction of next-generation sequencing, high-sensitivity genotyping, and publicly available high-coverage NGS data now allows accessing this region also in large genetic studies [17, 23, 57]. This has brought novel insights into the mechanisms by which SNPs regulate Lp(a) concentrations, such as the recent identification of two missense mutations (R1771, R990) that cause null alleles by impairing apo(a) folding and secretion [14] or the identification of a splice site variant (G4925A) that creates LMW apo(a) alleles with Lp(a) levels close to that of HMW alleles [23]. The latter has been recently used as a genetic instrument in a large study with > 140,000 individuals to separate the effects of the allele size (LMW/HMW) from the effects of Lp(a) concentrations [57]. These examples show that variants in the KIV-2 region can indeed provide new insights into the Lp(a) trait.

The *LPA* KIV-2 R21X variant is a nonsense mutation located in the KIV-2 region and results in a truncated protein, which is degraded quickly [13]. It is thus likely to causally determine Lp(a) concentrations and explain some peculiarities of the Lp(a) trait. Parson et al. [13] reported a MAF of 1.67% in Central Europeans (*n* = 405), but no study until now has investigated the contribution of R21X to the Lp(a) levels in populations nor it is known whether *LPA* KIV-2 R21X is in LD with any other SNP [24–26]. We have therefore developed an assay capable of typing R21X in large populations, typed in 10,910 Caucasian individuals and further assessed its frequency in 2504 individuals from 26 different populations of the 1000 Genomes study.

**Fig. 4:**
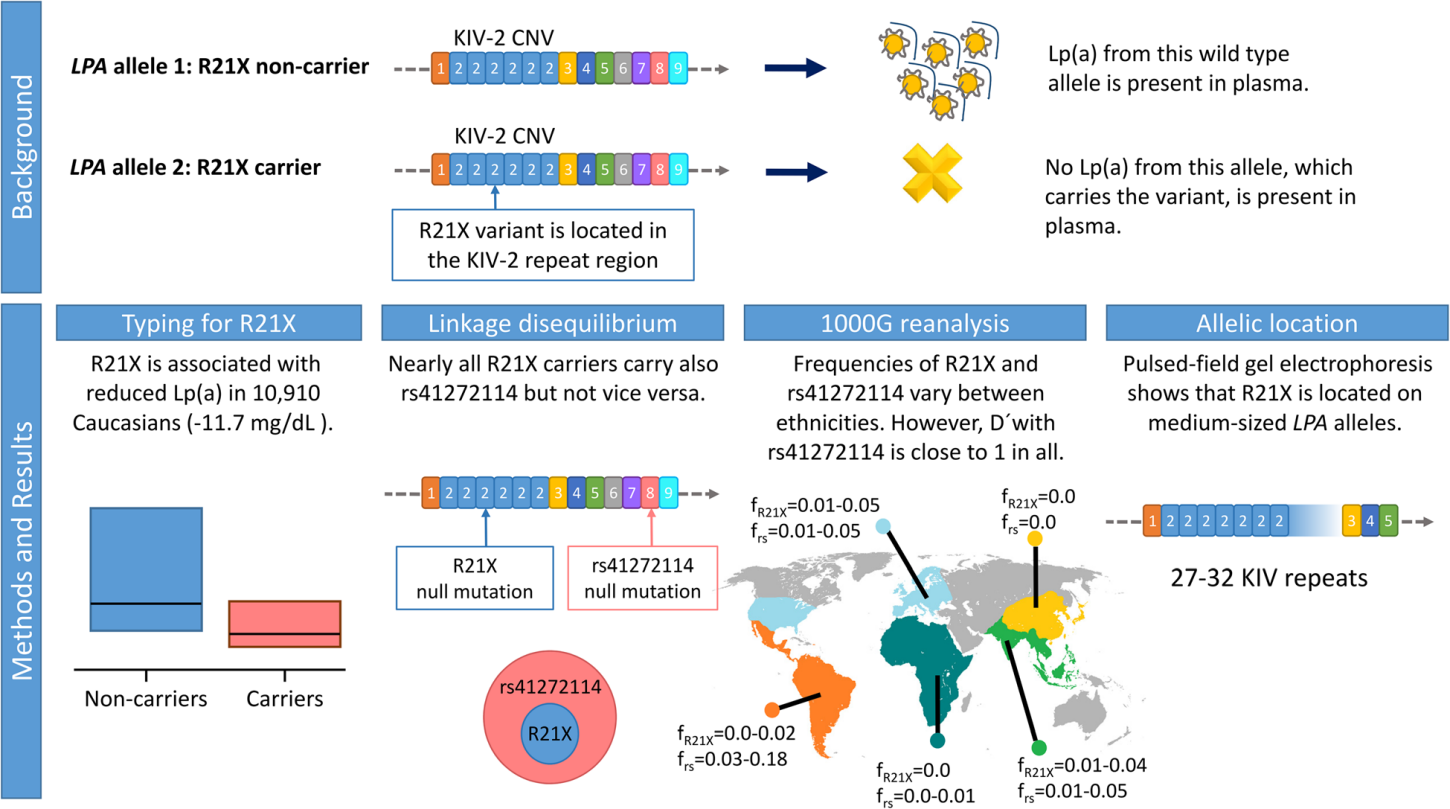
Graphical summary of the strategy and key findings of the study. *f*_R21X_: carrier frequency of R21X (heterozygous plus homozygous). *f*_rs_ = MAF ranges of rs41272114 in Ensembl 99 in the various 1000G populations

Our key experiments and findings are summarized in Fig. 4. We found that R21X is associated with a mean Lp(a) reduction of 9.9 to 13.0 mg/dL (Table 2). Considering that 10–12 mg/dL represents the median Lp(a) value in Caucasian populations, the effect size of R21X is conspicuous and another *LPA* loss-of-function variant that presents a similar effect size has been shown to be associated with reduced CVD risk [51, 52, 63]. Nevertheless, the effect size may appear still moderate for a nonsense mutation. This is explained by the observed location on moderate- to large-sized *LPA* alleles, which are per se associated with rather low Lp(a) concentrations. In comparison, the G4925A variant is located on isoforms 19–25 and thus associated with an Lp(a) reduction of ≈ 30 mg/dL [23]. R21X adds thus to the growing amount of examples of functional *LPA* SNPs that are confined to certain isoform ranges, which either masks [23, 61, 64], augments [23], or, like in the present work, limits their effects.

Surprisingly, the best proxy SNP for R21X among all hits of a recent genome-wide association meta-analysis on the Lp(a) concentrations [24] was rs41272114 (MAF = 2.6%). This SNP is a known splice site mutation in the KIV-8 domain of *LPA* and results in a null apo(a) allele, too [12, 51, 52]. Various studies have concordantly found a protective effect of this variant on CVD with odds ratios in the range of 0.71–0.85 [51, 52, 65]. The combination of a low determination coefficient (*R*^2^ = 0.27) but a high Lewontin’s *D*′ (*D*′ = 0.957) indicates that virtually all R21X carriers carry also rs41272114 (indicated by the high *D*′), but not vice versa (indicated by the low *R*^2^). This surprising localization on the same haplotype was confirmed also experimentally by PFGE. R21X is thus likely a more recent mutation that arose on the background of an rs41272114-carrying haplotype, and at least in the populations tested here, the R21X-carrying *LPA* haplotype is a subset of the more frequent rs41272114-carrying haplotypes. Therefore, despite the fact that R21X is a nonsense mutation and would be clearly functional in an isolated manner (e.g., in vitro), within its proper genomic context, its effect on Lp(a) is fully masked by rs41272114. Accordingly, we did not observe a significant difference between the median Lp(a) concentrations of individuals carrying only rs41272114 and individuals carrying both R21X and rs41272114. Only nine individuals carried R21X but not rs41272114, which is not sufficiently informative to compare the Lp(a) concentrations of R21X-only and rs41272114-only samples, given also the additional impact of the still functional second isoform.

The Lp(a) trait presents a pronounced interethnic variability [18], and differences in LD structures and/or SNP patterns have been described between populations [15, 62, 66]. We therefore investigated the LD structure of R21X and rs41272114 in the 1000G populations. R21X was largely restricted to Europeans and South Asians and absent in Africans, resembling the very low frequency of rs41272114 in Africans [16]. In Middle and South America (AMR group), R21X was present only in Colombians (CLM) and Puerto Ricans (PEL), with similar frequencies as in our own dataset. Conversely, the frequency of rs41272114 was highly population-specific, ranging from 0.03 in Colombians to 0.182 in Peruvians. The extraordinary high frequency of rs41272114 in the PEL population is of particular interest, since R21X is completely absent in this population. This indicates a very different LD structure of the *LPA* locus in the PEL group. However, *D*′ was still close to 1 also in AMR indicating that R21X is indeed located on the same haplotype as rs41272114 in all populations where it occurs, independently of frequency differences of rs41272114. It remains to be seen, whether populations exist (likely population isolates that have gone through a strong bottleneck in the past) where the pattern is reversed and R21X occurs independently from rs41272114. Rs41272114 has found to lower CVD risk [51, 52, 65]. If we assume that the effect in the population is conferred completely by the magnitude of Lp(a) lowering that is determined by the abolishing of the isoforms in the observed size range, also isolated R21X carriers may very likely benefit from the protective effect of this variant. Future studies searching for isolated R21X carriers may help to quantify the single effects of the two variants on Lp(a) levels and on CVD risk.

Our work has strengths and limitations. Our novel high-throughput ast-PCR capable of typing R21X, as well as G4925A, in a single multiplex reaction can be seen as a major technical strength of this work. Some commercial high-sensitivity assays like castPCR (Thermo Fisher Scientific), Agena MALDI-TOF Ultraseek [67], and droplet digital PCR [68] are able to type mutations in the KIV-2, too, but are too cost-intensive to be applied to large epidemiological studies. With nearly 11,000 individuals, the study at hand is the largest assessment of a KIV-2 variant performed so far.

Albeit it plays a major role whether a mutation is located on a low or a high molecular weight *LPA* allele, the allelic location of functional *LPA* mutations is rarely assessed in Lp(a) epidemiology. We demonstrated experimentally both that R21X is located on moderately large alleles and that R21X and rs41272114 form one haplotype. Conversely, the relatively low number of samples assessed by PFGE can be seen also as a limitation of our study. This is due to technical reasons. PFGE requires preparation of agarose plug-embedded DNA, which requires large amounts of buffy coat. This is not commonly available in typical epidemiological studies, and the low MAF of R21X further restricts the number of potential candidates for this experiment. Therefore, the size of our PFGE experiment is limited. Its results may thus not be fully generalizable and require follow-up investigations by independent studies. Still, the co-localization of rs41272114 and R21X on the same haplotype is currently supported by four complementary lines of evidence, three of them being independent from the PFGE experiment: (1) the experimental PFGE data; (2) the regression analysis in the GCKD dataset, where the effect of R21X, but not of rs41272114, vanishes after reciprocal adjustment; (3) the *D*′ values close to 1 in GCKD and KORA F3 and F4; and (4) the *D*′ close to 1 in thirteen ethnically diverse populations from the 1000G dataset.

Finally, we are aware that our association data is limited to individuals of European ancestry since no Lp(a) concentrations are available for the 1000G populations. Replication studies will be needed to assess the effects and LD patterns in other populations. Our data from the 1000G will hopefully help to select such studies.

We developed a high-throughput assay for the *LPA* KIV-2 variant R21X and found that this variant appears to be located on high molecular weight apo(a) alleles, lowers Lp(a) by 11.7 mg/dL, and most surprisingly, that it is in nearly complete LD with another null mutation (rs41272114). These two variants create *LPA* alleles that are inactivated by two independent loss-of-function mutations. Therefore, we show that, with respect to Lp(a) levels and cardiovascular risk, the R21X genotype does not provide additional information beyond the rs41272114 genotype. This has to be taken into account when using *LPA* loss-of-function mutations as instruments for genetic studies and emphasizes the complexity of *LPA* genetics.

## Supplementary information

**Additional file 1.** Supplementary methods and results. Contains the supplementary methods, the tables S1 to S9 and figures S1 to S5.

**Additional file 2.** Contains the rs41272114 genotypes and R21X carrier status for all 2504 individuals of the 1000 Genomes phase 3 v5 data at various sequencing coverage limits.

## Data Availability

The data from GKCD, KORA F3, and KORA F4 that support the findings of this study are available from the GCKD, respectively KORA steering committees but restrictions apply to the availability of these data (publication of individual-level datasets in public databases had not been a matter of the informed consent, and data access requires formal application to the steering committees). The data were used under license for the current study, and so are not publicly available. Data are however available from the corresponding author upon reasonable request and with permission from the respective steering committees. The 1000 Genome data analyzed in this study is freely available on the 1000 Genomes ftp data repository: ftp://ftp.1000genomes.ebi.ac.uk/vol1/ftp/phase3/data [45]. The individual-level results for 1000G newly generated in this study (i.e., R21X and rs41272114 genotypes, as well as sequencing coverage at the R21X locus) are provided in the Additional files 1 and 2 of this manuscript. The processing of this data was detailed in [17], and the analysis pipeline is provided in GitHub [48]. The qPCR data clustering script used for raw data processing is available in our GitHub repository [36].

## Abbreviations

apo(a): Apolipoprotein(a)
ast-PCR: Allele-specific TaqMan PCR
CAVASIC: Cardiovascular Disease in Intermittent Claudication (Study)
CKD: Chronic kidney disease
CKD-EPI: Chronic kidney disease epidemiology collaboration
CNV: Copy number variation
eGFR: Estimated glomerular filtration rate
GCKD: German Chronic Kidney Disease (Study)
GWAS: Genome-wide association studies
KORA: Cooperative Health Research in the Augsburg region (Study)
HMW: High molecular weight isoform
KIV: Kringle IV
LD: Linkage disequilibrium
LMW: Low molecular weight isoform
Lp(a): Lipoprotein(a)
MAF: Minor allele frequency
NGS: Next-generation sequencing
PFGE: Pulsed-field gel electrophoresis
SNP: Single nucleotide polymorphism
WT: Wild type
