# Supplementary material for: Investigation of a nonsense mutation located in the complex KIV-2 copy number variation region of apolipoprotein(a) in 10,910 individuals
Source: Genome Med. 2020 Aug 21;12:74. doi: 10.1186/s13073-020-00771-0 (PMC7442989; doi:10.1186/s13073-020-00771-0)
Supplement: Supplementary file 1 — Additional file 1. Supplementary methods and results. Contains the supplementary methods, the tables S1 to S9 and figures S1 to S5. [file 13073_2020_771_MOESM1_ESM.pdf]

Supplementary Materials to:

Investigation of a nonsense mutation located in the complex KIV-2 copy number variation region of apolipoprotein(a) in 10,910 individuals.

Silvia Di Maio[1], Rebecca Grüneis[1], Gertraud Streiter[1], Claudia Lamina[1], Manuel Maglione[2], Sebastian Schoenherr[1], Dietmar Öfner[2], Barbara Thorand[3], Annette Peters[3,4], Kai-Uwe Eckardt[5,6], Anna Köttgen[7], Florian Kronenberg[1], and Stefan Coassin[1]

[1] Institute of Genetic Epidemiology, Department of Genetics and Pharmacology, Medical University of Innsbruck, Innsbruck, Austria;
[2] Department of Visceral, Transplant and Thoracic Surgery, Medical University of Innsbruck, Innsbruck, Austria.
[3] Institute of Epidemiology, Helmholtz Zentrum München - German Research Center for Environmental Health (GmbH), Neuherberg, Germany
[4] German Centre for Cardiovascular Research (DZHK), Partner Site Munich Heart Alliance, Munich, Germany.
[5] Department of Nephrology and Hypertension, Friedrich-Alexander Universität Erlangen-Nürnberg, Erlangen, Germany
[6] Department of Nephrology and Medical Intensive Care, Charité—Universitätsmedizin Berlin, Berlin, Germany
[7] Institute of Genetic Epidemiology, Faculty of Medicine and Medical Center, University of Freiburg, Freiburg, Germany

Supplementary methods

Study populations

The GCKD (German Chronic Kidney Disease) study is an ongoing prospective observational cohort study[1] recruited at nine institutions in Germany. At the time of recruitment, participants were under nephrological care and presented moderate chronic kidney disease (CKD) from a broad spectrum of etiologies. No replacement therapies were required. Participants presented an estimated glomerular filtration rate (eGFR) of 30-60 mL/min × 1.73 m^2 or overt proteinuria in the presence of an eGFR>60 mL/min × 1.73 m^2. The calculation of the eGFR was done with the CKD-EPI equation based on creatinine values[2]. 5,049 DNA samples from the GCKD study were available at our institute. Apo(a) phenotypes, Lp(a) concentration, *LPA* genotyping for data for R21X and rs41272114 and remaining relevant variables were available for 4,771 individuals.

The KORA[3] (Cooperative Health Research in the Augsburg region, Kooperative Gesundheitsforschung in der Region Augsburg) cohorts F3 and F4 are representative for the general population in Augsburg and surrounding counties (Southern Germany) and are follow-up studies of the previous surveys KORA S3 and S4. These two non-overlapping surveys enrolled participants with German nationality and aged 25 to 74. KORA F3 was performed between 2004 and 2005 (n=3,184), while KORA F4 was performed between 2006 and 2008 (n=3,080). Measurements of the Lp-(a) levels and phenotyping of apo(a) isoforms were done in 3,156 participants of the KORA F3 subgroup and 3,061 subjects of the KORA F4 cohort. The DNA samples from the KORA study available at our institute were 3,161 from KORA F3 and 3,063 from KORA F4. Samples with available apo(a) phenotypes, Lp(a) concentration and *LPA* genotyping for the R21X were 3,099 for KORA F3 and 3,040 for KORA F4.

Samples for PFGE experiments

PFGE requires agarose-plug DNA preparation[5,6] from intact white blood cells. These were not available from the other studies above, because DNA had already been isolated and blood was not available anymore. Therefore we used for PFGE typing buffy coats from the Cardiovascular Disease in Intermittent Claudication (CAVASIC)[4] study and whole blood samples from an ongoing collection of liver tissue specimens for Lp(a) research (all of Caucasian ethnicity). Two additional samples (one R21X positive, one R21X negative) were selected from anonymous blood samples obtained from the blood bank of the University Hospital of Innsbruck, Austria. The CAVASIC study is a case-control study with a prospective follow-up investigation. Background and design of the study have been published earlier [4,7]. Briefly, 255 consecutive male patients with intermitted claudication (Fontaine Stage IIa or IIb regardless of whether they had already undergone a bypass surgery or intervention earlier) and

255 age- and diabetes-matched controls were enrolled. Nine individuals were positive for the R21X mutation. Buffy coats from all nine individuals were used for agarose-plug DNA preparation.

Liver tissue specimens were obtained from patients undergoing liver resection at the Department of Visceral, Transplant and Thoracic Surgery of the Medical University of Innsbruck. All patients undergoing conventional or laparoscopic, elective liver resection were considered for inclusion (provided written informed consent). Only patients with intact liver function or patients with chronic liver disease no worse than Child Pugh class A were included. One 2 cm^3 specimen from a macroscopically healthy portion of the resected liver was collected immediately after completion of the parenchymal resection, as well as two 9 ml EDTA-blood. One of the two EDTA blood tubes was then used to prepare agarose-plug DNA for PFGE analysis as described previously. Five samples (two R21X positive, three R21X negative) were selected for this study.

All twelve R21X carriers have been typed using KpnI-PFGE. In the Kpn2I-PFGE two samples did not show sufficient separation between the two alleles to be excised from the agarose gel for subsequently typing of R21X on the isolated alleles. Ten samples were successfully typed for R21X and rs41272114 on the isolated gene alleles using Kpn2I.

Site-directed mutagenesis

The R21X variant was introduced by site-directed mutagenesis following the instruction manual of the QuikChange II Site-Directed Mutagenesis Kit (Agilent Technologies, Santa Clara, CA, USA) with some modifications. We used the KAPA High Fidelity Polymerase (KAPA Biosystem, Wilmington, MA, USA) for the mutant strand synthesis. Primer sequences are given in Table S1. Details about the PCR protocols and primers are given in Table S9. The parental strand was digested with DpnI and transformation was done following the High Efficiency Transformation Protocol of New England Biolabs (NEB, Ipswich, Massachusetts, USA) in NEB 10-beta E. coli. GeneJET Plasmid Miniprep Kit (ThermoFisher Scientific, Waltham, Massachusetts, USA) was used for plasmid isolation.

ast-PCR efficiency and quality control

Assay performance

1. Six dilutions 1:10 ranging from 138 pg/µl to 1.38 fg/µl of the mutant plasmid (pSPL3 carrying the *LPA* fragment mutant for the R21X) were used for qPCR efficiency testing. The DNA inputs used for ast-PCR are 20 to 40 ng, which correspond to 6,060 and 12,120 human genome copies. Therefore, an equimolar amount of plasmid was used as starting point of the dilution series (138 fg, corresponding to 12,120 plasmid copies). Amplification efficiency was 98.63%.
2. Test on plasmid dilution to assess the lowest fraction of mutant detectable. Our ast-PCR enabled the detection of a fractional representation of the mutant allele of down to 0.5% (test performed on pSPL3, Figure S2). In the genomic DNA, the lowest expected mutation level is ≈1.2% (1 mutant KIV-2 in ≈80 wild type KIV-2).

The validation steps adopted were:

1. Validation of the ast-PCR against the commercial cast-PCR (ThermoFisher Scientific) used by Coassin and colleagues[8]: For the G4925A probe, we compared the results of 376 samples from KORA F4 with the results previously obtained by Coassin et al [8]. Subsequently, during study data generation we genotyped the whole KORA F4 population again and compared the results with the commercial castPCR used in Coassin et al, 2019[8]. For the R21X[9] probe, we run our assay on 376 samples from KORA F4. All 14 samples identified as R21X carriers by our assay were confirmed also by a commercial castPCR assay with a declared sensitivity of 0.2% mutant fraction. Results are reported in Table S5.
2. Reproducibility was tested by genotyping 477 samples in duplicates (n_{total}=954; GCKD: n=267, 5.4%; KORA F3: n=298, 9.44%; KORA F4: n=314, 10.35%). The discordance rate was 0% (Table S5).
3. False negative results due to PCR failure was controlled by inclusion of a positive amplification control in *PNPLA3* in each reaction.
4. A positive control sample (R21X carrier status determined with the commercial castPCR) was included on each 384 well qPCR plate (34 plates). The ast-PCR provided consistent results in all 34 384-well plates that were run.
5. The call rate (defined as the ratio between the called samples (samples whose carrier status could be unambiguously determined) and the total number of DNA samples typed), during study data generation ranged from 97.8% to 99.0% (Table S5).

ast-PCR data analysis

Samples were classified as carriers or non-carriers using a statistical two-step approach (implemented in R) to avoid human bias when assessing the amplification curves. In the first step, an optimal discrimination threshold between the carriers and non-carriers is estimated using a bagged clustering algorithm[10] implemented in the function 'classIntervals' (package 'classInt' available at https://CRAN.R-project.org/package=classInt).

In the second step, the parameters of a univariate normal distribution are estimated via Maximum likelihood (function 'vglm', package 'VGAM')[11] using all Ct-values below the obtained optimal threshold for the carrier distribution and above the threshold for the non-carrier distribution. Values below the threshold are classified as carriers, above as non-carriers. However, values that cannot be assigned unambiguously to one of the two distributions are marked as outliers.

All Ct-values above the 97.5% percentile of the carrier and 2.5% percentile of the non-carrier distribution were marked in this analysis and were excluded from analysis. The rate of samples excluded from analysis was 0.7% in GCKD (35/4974), 1.6% in KORA F3 (52/3157) and 0.5% in KORA F4 (15/3063). The custom R function used for analysis is available at https://github.com/genepi/r21x_analysis.

ast-PCR for PFGE alleles

To determine the allelic location of R21X we run a modified protocol of the newly established ast-PCR on the extracted DNA from PFGE. Due to the variable amount of DNA extracted from the agarose plugs for PFGE, the Ct of positive samples can vary widely and no clear distribution of Ct values as in gDNA from population studies can be expected. Therefore, each sample was run both with a R21X wild type and a R21X mutant-specific primer and the R21X wild type specific reaction was used as reference signal that defines the amount of DNA present in the plug. Samples where $Ct_{\_mut}$-$Ct_{\_WT}$ was ≤6.5 (i.e. ≥1% relative abundance of the mutant) were classified as R21X carriers. A general primer for the G4925A A-allele was added to keep the number of oligos in the reaction unchanged compared to the population-screening assay but was not evaluated further. The approach using both primers is detailed in [8].

Supplementary tables

Table S1. Sequences of primers and probes.

Target	Function	Sequence 5´-3´	Labels
**ast-PCR for population genotyping**			
G4925A-carrier *LPA* (108 bp)	ASP primer	CTAGAGGCTCCTTCCGAACATA	
	Second primer	ATTGAAGGCGGCTGCATCAG	
	Probe	CTGTGGCCAGACATCTACACGCTTCGA	5’ FAM, 3´ BHQ2
R21X-carrier *LPA* (182 bp)	ASP primer	TGACAGTGGTGGAGTATGTGCCTAA	
	Second primer	AATTCTCATAGACTCCTTTCTGGTTGTGTC	
	Probe	TTCCCGTGTTTTCATTTCAGCACCG	5´ATTO550, 3´BHQ2
Control fragment (*PNPLA3*) (130 bp)	Fw primer	AATGCCTCCCGGCCAAT	
	Rv primer	CTGCTTACATCCACGACTTCGT	
	Probe	TCCACCAGCTCATCTCCGGCAAA	5´ YAKIMA YELLOW, 3´ BHQ1
**rs41272114 sequencing**			
rs41272114	Fw PCR primer	GGGTTCAGGACTGCTACCGA	
	Rv PCR primer	CTTTAGTGGGCATTGTGGAATGATAC	
	Sequencing primer	GGGTTCAGGACTGCTACCGA	

(continues)

(Table S1, continued)

Target	Function	Sequence 5´-3´	Labels
**ast-PCR for allele localization in PFGE alleles ***			
G4925A *LPA*	First primer	CTAGAGGCTCCTTCCGAACAAA	
(108 bp)	Second primer	ATTGAAGGCGGCTGCATCAG	
	Probe	CTGTGGCCAGACATCTACACGCTTCGA	5’ FAM, 3´ BHQ2
R21X WT *LPA*	ASP primer	TGACAGTGGTGGAGTATGTGCCTTG	
(182 bp)	Second primer	AATTCTCATAGACTCCTTTCTGGTTGTGTC	
	Probe	TTCCCGTGTTTTCATTTCAGCACCG	5´ATTO550, 3´BHQ2
**Plasmid mutagenesis**			
Mutagenesis	Fw R21X mutagenic primer	TACCATGGTAATGGACAGAGTTATTGAGGCACATACTC	
	Rv R21X mutagenic primer	GAGTATGTGCCTCAATAACTCTGTCCATTACCATGGTA	
Confirmatory sequencing	Fw PCR primer[5]	AGAAACAAACCTACTAAACCTGACAG	
	Rv PCR primer[5]	TTTTTCTGACAATCGGAATATAC	
	Fw sequencing primer[5]	TTGGCTTTCATGATCAACG	

SNP base: underlined. Additional mismatch base: red letter[12]. Fw: forward, Rv: reverse, BHQ: black hole quencher, WT: wild type, ASP: allele-specific primer, bp: base pair. All probes were ordered from Eurofins Genomics (Ebersberg, Germany).

* Only primers for the wild type alleles reported. The primers for the mutant allele were the same as used for population screening.

Table S2. ast-PCR protocol.

Step	Setting
PCR master mix	Agilent Brilliant III Ultra Fast qPCR<br>2x Master Mix
Targets	G4925A-carrier *LPA* fragment (108 bp)<br>R21X-carrier *LPA* fragment (182 bp)<br>Control fragment (*PNPLA3*) (130 bp)
Reaction volume [µl]	5
Primer concentration [µM each] *	G4925A: 0.3, R21X: 0.3, PNPLA3: 0.1
Probe concentration [µM] *	0.2
Master Mix amount [µl]	2.5
DNA input (ng/reaction)	20
**Cycling conditions**	
UDG activation	50°C, 2 min
Polymerase Activation	95°C, 3 min
Denaturation	95°C, 15 s
Annealing/extension	60°C, 1 min
Number of cycles	40

Cycling was done on a ThermoFisher QuantStudio 6 qPCR system with 384-well block.
* Probes and primer sequences are given in Table S1.

Table S3. PFGE protocol (Bio-Rad Chef Mapper System)

	KpnI	Kpn2I
Gel concentration	1.0%	1.8%
Running buffer	0.5X TAE	0.5X TBE
Run duration	18 hrs 23 min	57 hrs 2 min
Temperature	14°C	14°C
Included angle	120°	120°
Initial switch time	1.73 s	1 min 1.61 s
Final switch time	14.92 s	1 min 43.15 s
Gradient	6	6
Ramping constant	0	-1.340

Table S4. PCR protocol for sequencing rs41272114 on genomic DNA.

For sequencing on isolated PFGE alleles the protocol was run as nested PCR protocol, where the same protocol was re-run on a 1 µl aliquot of the first PCR on the PFGE alleles.

Step	Setting
Product length	448 bp
Reaction volume [µl]	10
Enzyme	HotStarTaq DNA Polymerase 5 U/µl
Initial denaturation	94°C, 15 min
Denaturation	94°C, 30 s
Annealing	60°C, 30 s
Extension	72°C, 30 s
Final extension	70°C, 10 min
Number of cycles	40
Primer fw *	LPA_rs41272114_f2
Primer rv*	LPA_rs41272114_r1
Final primer concentration [µM each]	0.25
Final dNTP concentration [mM each]	0.25
Enzyme amount [U]	0.25
DNA input [ng]	20

Sequencing was done on an ABI 3130xl system using ThermoFisher Scientific BigDye v1.1 chemistry.
Fw: forward, Rv: reverse
* Primer sequences are given in Table S1.

Table S5. Genotyping quality measures.

Study	GCKD	KORA F3	KORA F4
n samples	5049	3161	3063
n called samples	4939	3105	3048
Call rate	97.82%	98.23%	99.00%
n QC samples	275	308	317
n called QC samples	267	298	314
% called QC samples	5.4	9.56	10.30
n discordant samples	0	0	0
Discordance rate	0.0%	0.0%	0.0%

QC: quality control samples (samples present in duplicates); n: number.

Table S6. PFGE genotypes and R21X carrier status.

Sample	*LPA* genotype (KpnI)	Allele carrying the R21X variant (Kpn2I)	Allele carrying the rs41272114 variant (Kpn2I)	rs41272114 genotype (gDNA)
**Results from Kpn2I and KpnI PFGE for R21X and rs41272114 (haplotyping)**				
sample #1	21/32	32	32	CT
sample #2	28/28	28	28	CT
sample #3	31/31	31	31	TT
sample #4	22/30	30	30	CT
sample #5	23/30	30	30	CT
sample #6	24/27	27	27	CT
sample #7	24/30	30	30	CT
sample #8	30/30	30	30	CT
sample #9	28/33	33	33	CT
sample #10	21/28	28	28	CT
sample #11*	24/27	27	NA†	CT
sample #12*	32/32	32	NA†	CT
NC #1[×]	29/39	WT ‡‡	29	CT
NC #2*	26/26	no amp. ‡	no amp. ‡	CC
NC #3*	27/29	no amp. ‡	no amp. ‡	CC
NC #4*	24/31	no amp. ‡	no amp. ‡	CC

Selected samples carrying the R21X mutation and the rs41272114 were subjected to PFGE and the separated alleles were typed for R21X and rs41272114. No ast-PCR amplification was seen in any of the alleles of the control samples (NC #1 to #4). Rs41272114 genotypes are given relative to the plus strand of the human genome reference hg38 (T is the minor allele causing the splicing defect). WT: wild type

* Samples were determined only by KpnI-PFGE due to insufficient separation in the Kpn2I-PFGE.

† Allele-specific genotype could not be determined because the locus of rs41272114 is not present in the KpnI-PFGE fragment.

× Sample is R21X negative but rs41272114 positive.

‡ no amp.: no amplification. Samples are wild type for both SNPs. As expected the samples did not show any amplification in both assays (negative control sample).

‡‡ Sample is wild type for R21X. As expected, the sample did not show any amplification in the R21X assays while being heterozygous for rs41272114 (negative control sample).

Table S7. Frequency of R21X in the single populations of the 1000 G high coverage exome dataset at no lower coverage limit, 340X and 780X.

Super pop.	AFR							AMR				EAS					EUR					SAS				
**Population**	**ACB**	**ASW**	**ESN**	**GWD**	**LWK**	**MSL**	**YRI**	**CLM**	**MXL**	**PEL**	**PUR**	**CHB**	**CHS**	**CDX**	**KHV**	**JPT**	**CEU**	**FIN**	**GBR**	**IBS**	**TSI**	**BEB**	**ITU**	**GIH**	**PJL**	**STU**
**All**	**n=661**							**n=347**				**n=504**					**n=503**					**n=489**				
**Individuals [n]**	96	61	99	113	99	85	108	94	64	85	104	103	105	93	99	104	99	99	91	107	107	86	102	103	96	102
**Carriers [n]**	0	0	0	0	0	0	0	1	0	0	2	1	0	0	0	0	5	2	2	1	2	3	1	1	3	1
**Carrier freq.**	0	0	0	0	0	0	0	0.011	0	0	0.019	0.010	0	0	0	0	0.051	0.020	0.022	0.009	0.019	0.035	0.010	0.010	0.031	0.010
**Coverage >340**	**n=561**							**n=300**				**n=414**					**n=362**					**n=468**				
**Individuals [n]**	77	52	99	113	41	85	94	77	56	63	104	78	98	77	79	81	65	86	68	68	73	86	102	82	96	102
**Carriers [n]**	0	0	0	0	0	0	0	1	0	0	2	0	0	0	0	0	3	2	2	1	2	3	1	1	3	1
**Carrier freq.**	0	0	0	0	0	0	0	0.013	0	0	0.019	0	0	0	0	0	0.046	0.023	0.029	0.015	0.027	0.035	0.010	0.012	0.031	0.010
**Coverage >780**	**n=548**							**n=279**				**n=333**					**n=277**					**n=468**				
**Individuals [n]**	73	52	99	113	38	85	88	69	56	61	93	55	60	69	71	77	61	42	50	52	67	86	102	82	96	102
**Carriers [n]**	0	0	0	0	0	0	0	1	0	0	2	0	0	0	0	0	3	1	1	0	2	3	1	1	3	1
**Carrier freq**	0	0	0	0	0	0	0	0.014	0	0	0.022	0	0	0	0	0	0.049	0.024	0.020	0	0.030	0.035	0.010	0.012	0.031	0.010

The row “individuals [n]” reports the number of individuals reaching the given coverage at the R21X location. Super pop.: 1000 G super population (continental group); Carrier freq.: Carrier frequency; AFR: Africans; AMR: Ad-mixed Americans; EAS: East Asians; EUR: Europeans; SAS: South Asians; n: number. See Table S8 for the meaning of the population codes.

Table S8. Frequency of rs41272114 in the single populations of the 1000G high coverage exome dataset compared to the frequency of R21X (all, and >780X coverage only).

		rs41272114		R21X			
**Pop**	**Population description**	**MAF**	**MAF GnomAD**	**Carrier freq.**	**n_C**	**Carrier freq. >780X**	**Carriers >780X [n]**
**AFR**	**African**	**0.002**	**0**	**0**	**0**	**0**	**0**
ACB	African Caribbeans in Barbados	0.005	-	0	0	0	0
ASW	Americans of African Ancestry in SW USA	0.008	-	0	0	0	0
ESN	Esan in Nigeria	0	-	0	0	0	0
GWD	Gambian in Western Divisions in the Gambia	0	-	0	0	0	0
LWK	Luhya in Webuye, Kenya	0.005	-	0	0	0	0
MSL	Mende in Sierra Leone	0	-	0	0	0	0
YRI	Yoruba in Ibadan, Nigeria	0	-	0	0	0	0
**AMR**	**Ad Mixed American**	**0.078**	**0.104**	**0.009**	**3**	**0.011**	**3**
CLM	Colombians from Medellin, Colombia	0.032	-	0.011	1	0.014	1
MXL	Mexican Ancestry from Los Angeles USA	0.086	-	0	0	0	0
PEL	Peruvians from Lima, Peru	0.182	-	0	0	0	0
PUR	Puerto Ricans from Puerto Rico	0.029	-	0.019	2	0.022	2
**EAS**	**East Asian**	**0**	**0.001**	**0.002**	**1**	**0**	**0**
CDX	Chinese Dai in Xishuangbanna, China	0	-	0	0	0	0
CHB	Han Chinese in Bejing, China	0	-	0.010	1	0	0
CHS	Southern Han Chinese	0	-	0	0	0	0
KHV	Kinh in Ho Chi Minh City, Vietnam	0	-	0	0	0	0
JPT	Japanese in Tokyo, Japan	0	-	0	0	0	0
**EUR**	**European**	**0.031**	**0.036**	**0.024**	**12**	**0.026**	**7**
CEU	Utah Residents (CEPH) with Northern and Western Ancestry	0.051	-	0.051	5	0.049	3
FIN	Finnish in Finland	0.035	-	0.020	2	0.024	1
GBR	British in England and Scotland	0.011	-	0.022	2	0.020	1
IBS	Iberian Population in Spain	0.037	-	0.009	1	0	0
TSI	Toscani in Italia	0.019	-	0.019	2	0.030	2

		rs41272114		R21X			
**Pop**	**Population description**	**MAF**	**MAF GnomAD**	**Carrier freq.**	**n_C**	**Carrier freq. >780X**	**Carriers >780X [n]**
**SAS**	**South Asian**	**0.030**	**0.034**	**0.019**	**9**	**0.019**	**9**
BEB	Bengali from Bangladesh	0.047	-	0.035	3	0.035	3
GIH	Gujarati Indian from Houston, Texas	0.019	-	0.010	1	0.012	1
ITU	Indian Telugu from the UK	0.025	-	0.010	1	0.010	1
PJL	Punjabi from Lahore, Pakistan	0.052	-	0.031	3	0.031	3
STU	Sri Lankan Tamil from the UK	0.010	-	0.010	1	0.010	1

For comparison also the MAF of rs41272114 in GnomAD[13] (GnomAD group “Latino”). The frequency for EUR consists of the combined GnomAD frequencies for all Europeans (Finns and non-Finns). Pop.: 1000 G population; Carrier freq.: carrier frequency. n_C: number of carriers

Table S9. Site-directed mutagenesis PCR protocol.

	Site-directed mutagenesis
Plasmid length	10422
Reaction volume [µl]	30
Enzyme	Kapa Biosystems KAPA HiFi, 1 U/µl
Initial denaturation	95°C, 3 min
Denaturation	98°C, 20 s
Annealing	66°C, 15 s
Extension	72°C, 5 min
Final extension	72°C, 10 min
Number of cycles	12
Final primer concentration [µM each]	0.3
Final dNTP concentration [mM each]	0.3
Enzyme amount [U]	0.5
DNA input [ng]	15

Primer sequences are given in Table S1.

Supplementary figures

Fig. S1. Assay design

This figure illustrates the triplex ast-PCR assay design. Blue character: target bases. Each allele-specific primer includes a mismatch at the penultimate primer position (read 5' to 3'). The stars indicate the different Taqman labels (red: ATTO550. green: FAM, light blue: Yakima Yellow) and the circles the 3' black hole quenchers of the TaqMan probes. The allele-specific primer for R21X targets the mutant base on the minus strand of the genome hg38, while the G4925A assay targets the plus strand.

Since the amount of DNA retrieved from the agarose blocks excised from the PFGE varies largely, for the PFGE-isolated alleles sample was run also with primers specific for the wild type base, which served as reference Ct value to determine the amount of DNA present in the agarose isolate.

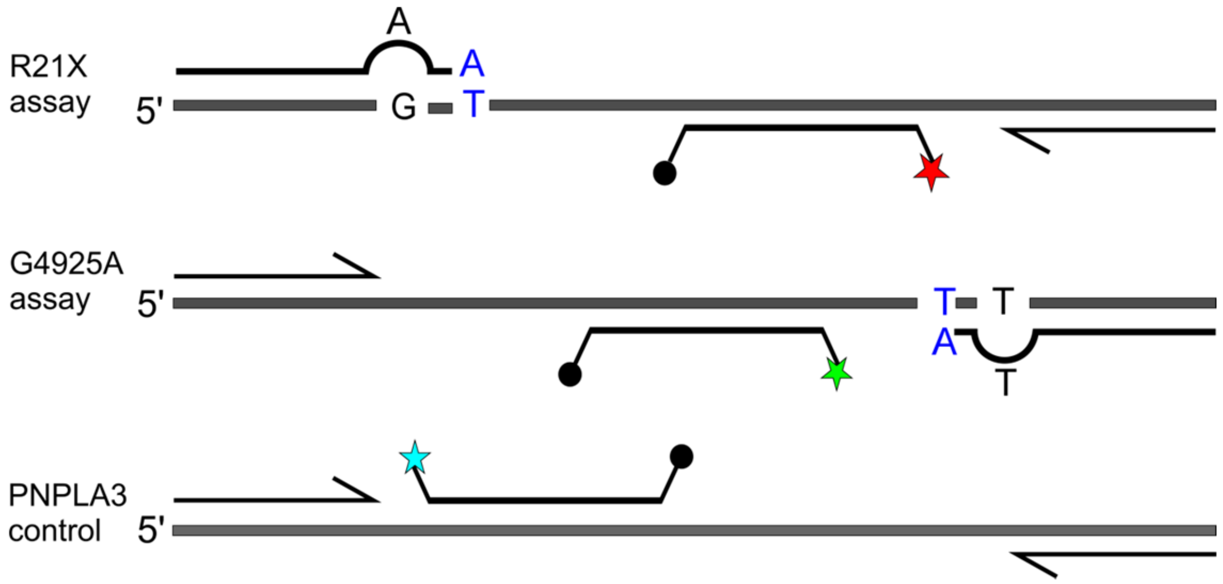

Fig. S2. Dilution experiment for the R21X assay.

The mutant pSPL3 plasmid containing the R21X mutation (see methods in the main document) has been diluted into a wild type pSPL3 in varying percentage. 0% corresponds to wild type plasmid only. A clear unique amplicon is still observed at 0.5% mutation level, but no amplicon is observed at 0% mutation level. Also in a qPCR setting 0.5% could be clearly discriminated from 0% as the average Ct value (triplicates) was 33.6 for the 0.5% plasmid mix and >40 ("undetermined") for the 0% mix. Top: inverted image. Bottom: standard image. The percentage of mutant plasmid in wild type plasmid is given above each sample pair. For each sample pair, the annealing was performed at 60°C for the left band and at 61.5°C for the right band. For all subsequent experiments 60°C annealing temperature was used.

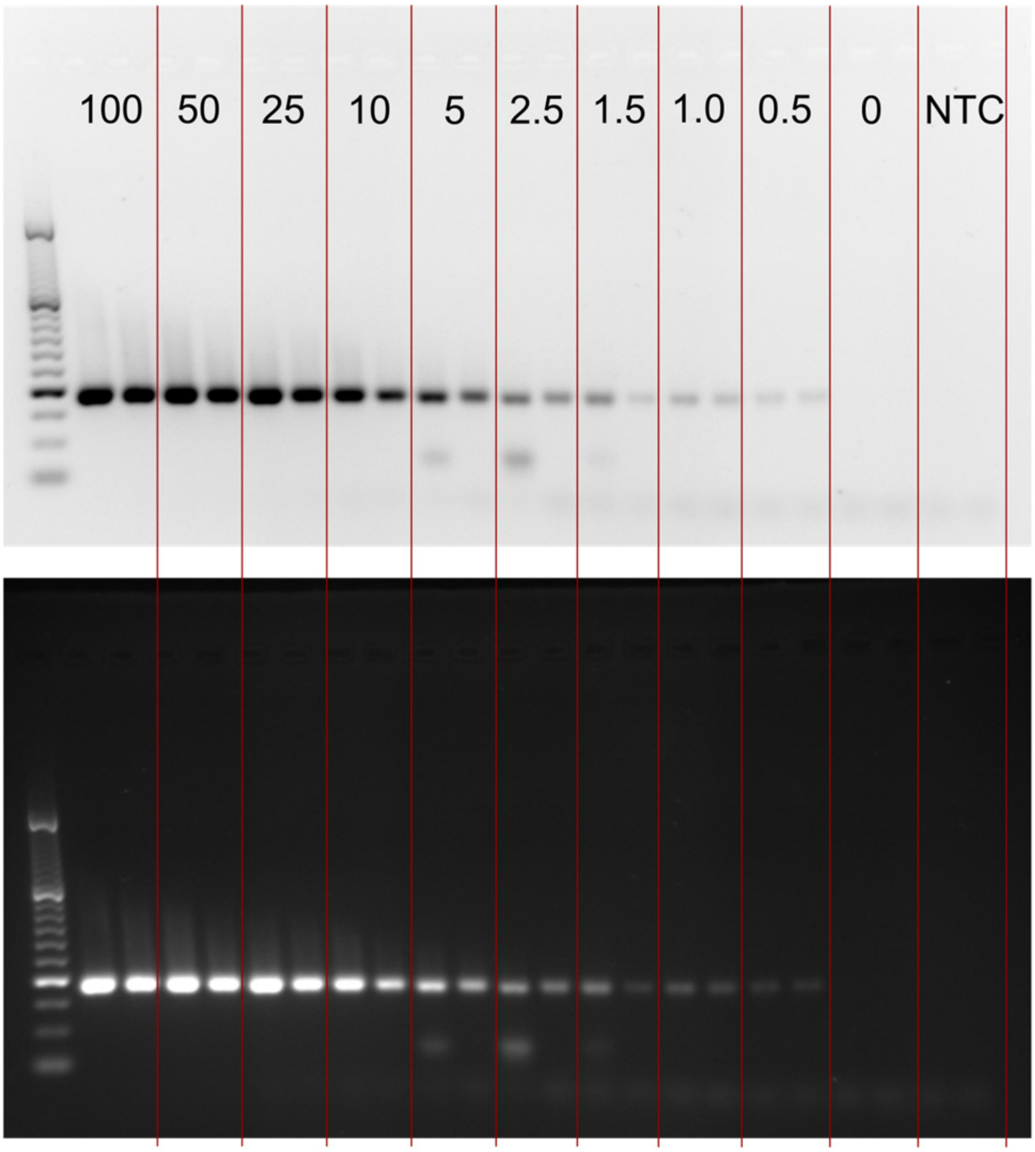

Fig. S3. Genomic region of LPA.

The region excised by Kpn2I digestion is highlighted in yellow, while the black annotated line ("KpnI fragment") indicates the region excised by KpnI digestion. Red: location of rs41272114; orange: location of the KIV-2 repeat. Note that *LPA* is encoded on the minus strand. Region shown: hg19, chr6:160,803,579-161,499,145 (from the UCSC Genome Browser).

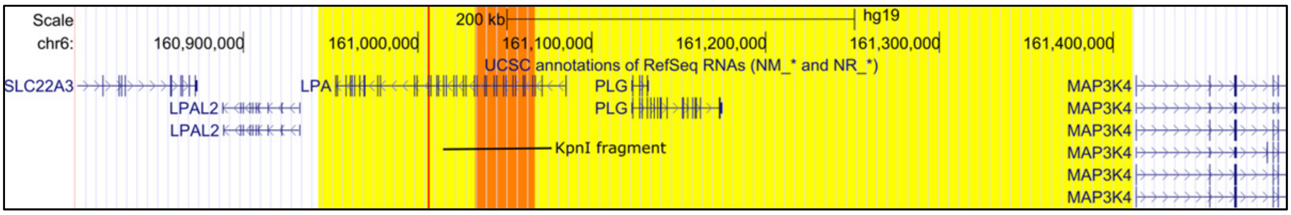

Fig. S4. Mutation level of R21X in the 1000G super populations.

The mutation level represents the fractional proportion of KIV-2 repeats carrying the mutation in the respective individual. The maximum level (9.5%) was seen in EUR individual HG00284. No carrier was observed in Africans (AFR). Error bars represent mean ± standard deviation. Data without coverage filtering was used for plotting. Each dot represents an 1000G individual.

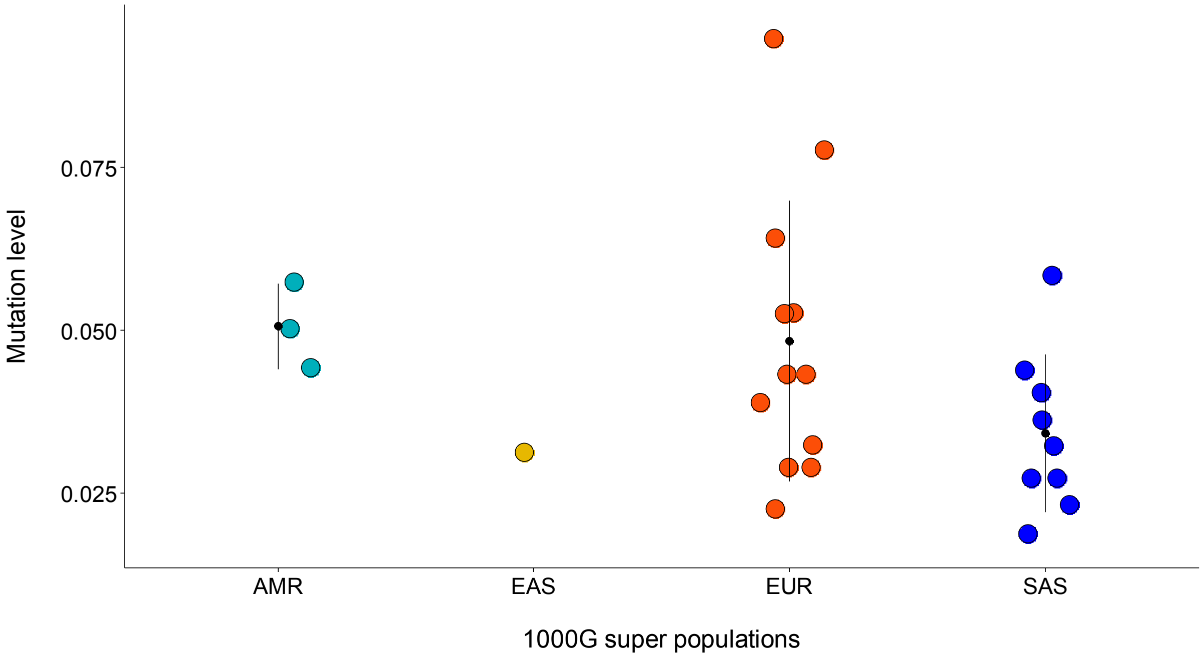

Fig. S5. Association of the R21X variant with reduced Lp(a) levels.

Lp(a) is lower in R21X variant carriers in each population. Lp(a) concentration is expressed in mg/dL.

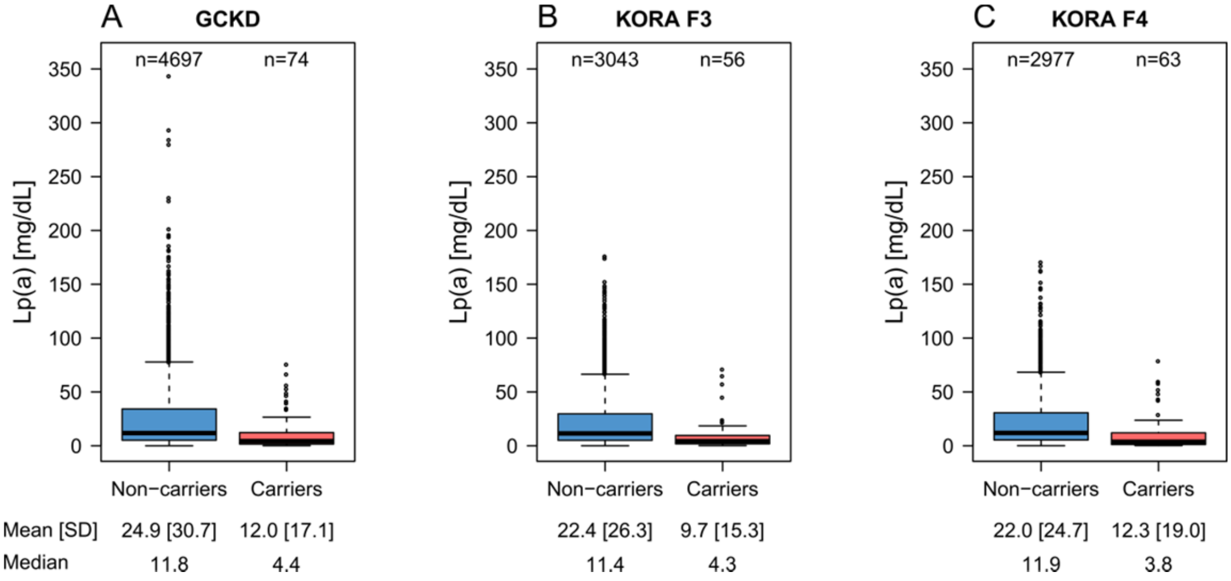

References

1. Titze S, Schmid M, Köttgen A, Busch M, Floege J, Wanner C, et al. Disease burden and risk profile in referred patients with moderate chronic kidney disease: Composition of the German Chronic Kidney Disease (GCKD) cohort. Nephrol Dial Transplant. 2015;30:441–51.
2. Levey AS, Stevens LA, Schmid CH, Zhang YL, Castro AF, Feldman HI, et al. A New Equation to Estimate Glomerular Filtration Rate. Ann Intern Med. 2009;150:604.
3. Wichmann H-E, Gieger C, Illig T, MONICA/KORA Study Group. KORA-gen--resource for population genetics, controls and a broad spectrum of disease phenotypes. Gesundheitswesen. 2005;67 Suppl 1:S26-30.
4. Rantner B, Kollerits B, Anderwald-Stadler M, Klein-Weigel P, Gruber I, Gehringer A, et al. Association between the UGT1A1 TA-repeat polymorphism and bilirubin concentration in patients with intermittent claudication: results from the CAVASIC study. Clin Chem. 2008;54:851–7.
5. Noureen A, Fresser F, Utermann G, Schmidt K. Sequence Variation within the KIV-2 Copy Number Polymorphism of the Human LPA Gene in African, Asian, and European Populations. PLoS One. 2015;10:e0121582.
6. Kraft HG, Köchl S, Menzel HJ, Sandholzer C, Utermann G. The apolipoprotein (a) gene: a transcribed hypervariable locus controlling plasma lipoprotein (a) concentration. Hum Genet. 1992;90:220–30.
7. Vogl L, Pohlhammer J, Meinitzer A, Rantner B, Stadler M, Peric S, et al. Serum concentrations of L-arginine and L-homoarginine in male patients with intermittent claudication: a cross-sectional and prospective investigation in the CAVASIC Study. Atherosclerosis. 2015;239:607–14.
8. Coassin S, Erhart G, Weissensteiner H, Eca Guimarães de Araújo M, Lamina C, Schönherr S, et al. A novel but frequent variant in LPA KIV-2 is associated with a pronounced Lp(a) and cardiovascular risk reduction. Eur Heart J. 2017;38:1823–31.
9. Parson W, Kraft HG, Niederstätter H, Lingenhel AW, Köchl S, Fresser F, et al. A common nonsense mutation in the repetitive Kringle IV-2 domain of human apolipoprotein(a) results in a truncated protein and low plasma Lp(a). Hum Mutat. 2004;24:474–80.
10. Leisch F. Bagged clustering. Working Papers SFB “Adaptive Information Systems and Modelling in Economics and Management Science”, 51. SFB Adaptive Information Systems and Modelling in Economics and Management Science, WU Vienna University of Economics and Business, Vienna; 1999.
11. Yee TW. The VGAM Package for Categorical Data Analysis. J Stat Softw. 2010;32:481–93.
12. You FM, Huo N, Gu YQ, Luo M-C, Ma Y, Hane D, et al. BatchPrimer3: a high throughput web application for PCR and sequencing primer design. BMC Bioinformatics. 2008;9:253.
13. Karczewski KJ, Francioli LC, Tiao G, Cummings BB, Alföldi J, Wang Q, et al. Variation across 141,456 human exomes and genomes reveals the spectrum of loss-of-function intolerance across human protein-coding genes. bioRxiv. 2019;531210.
